# Facile In Situ Synthesis of ZnO Flower-like Hierarchical Nanostructures by the Microwave Irradiation Method for Multifunctional Textile Coatings

**DOI:** 10.3390/nano11102574

**Published:** 2021-09-30

**Authors:** Maria Antonia Tănase, Andreia Cristina Soare, Petruţa Oancea, Adina Răducan, Cătălin Ionuţ Mihăescu, Elvira Alexandrescu, Cristian Petcu, Lia Mara Diţu, Marilena Ferbinteanu, Bogdan Cojocaru, Ludmila Otilia Cinteza

**Affiliations:** 1 Physical Chemistry Department, Faculty of Chemistry, University of Bucharest, 4-12 Regina Elisabeta Blv., 030018 Bucharest, Romania; maria.a.tanase@gmail.com (M.A.T.); andreia-cristina.soare@drd.unibuc.ro (A.C.S.); petrutaoancea73@yahoo.com (P.O.); adina.raducan@g.unibuc.ro (A.R.); 2 Polymers Department, National Institute for Research and Development in Chemistry and Petrochemistry—ICECHIM, 202 Spl. Independentei, 060021 Bucharest, Romania; mihaescu_catalin96@yahoo.com (C.I.M.); elviraalexandrescu@yahoo.com (E.A.); 3 Microbiology Department, Faculty of Biology, University of Bucharest, 1-3 Aleea Portocalilor, 060101 Bucharest, Romania; lia-mara.ditu@bio.unibuc.ro; 4 Inorganic Chemistry Department, Faculty of Chemistry, University of Bucharest, 23 Dumbrava Rosie, 020462 Bucharest, Romania; marilena.cimpoesu@g.unibuc.ro; 5 Organic Chemistry, Biochemistry and Catalysis Department, Faculty of Chemistry, University of Bucharest, 4-12 Regina Elisabeta Blv., 030018 Bucharest, Romania; bogdan.cojocaru@chimie.unibuc.ro

**Keywords:** ZnO nanoparticles, in situ synthesis, microwave synthesis, functional textiles, antimicrobial textile

## Abstract

ZnO nanoparticle-based multifunctional coatings were prepared by a simple, time-saving microwave method. Arginine and ammonia were used as precipitation agents, and zinc acetate dehydrate was used as a zinc precursor. Under the optimized conditions, flower-like morphologies of ZnO aggregates were obtained. The prepared nanopowders were characterized using X-ray powder diffraction (XRD), scanning electron microscopy (SEM), and UV/Visible spectroscopy. The developed in situ synthesis with microwave irradiation enabled significant ZnO nanoparticle deposition on cotton fabrics, without additional steps. The functionalized textiles were tested as a photocatalyst in methylene blue (MB) photodegradation and showed good self-cleaning and UV-blocking properties. The coated cotton fabrics exhibited good antibacterial properties against common microbial trains (*Staphylococcus aureus*, *Escherichia coli,* and *Candida albicans*), together with self-cleaning and photocatalytic efficiency in organic dye degradation. The proposed microwave-assisted in situ synthesis of ZnO nanocoatings on textiles shows high potential as a rapid, efficient, environmentally friendly, and scalable method to fabricate functional fabrics.

## 1. Introduction

The variety of metal oxides is wide, and their range of properties and possible applications appear to be enormous. Zinc oxide (ZnO) is a multifunctional material due to its exceptional physicochemical properties and broad usefulness, such as in corrosion protection [1], gas sensors [2], pollutants sorbents [3,4], food packaging [5], piezoelectric devices [6], electronics [7], agriculture [8], functional textiles [9], and photocatalysts for organic waste decomposition [10]. Other applications of zinc oxide nanopowders include electrophotography, photoprinting, capacitors, protective coatings, anti-microbial agents, and conductive thin-films in LCDs, solar cells, and blue laser diodes [11].

Optoelectronic applications of ZnO nanoparticles (ZnO NPs) are based on their emissions, one in the ultraviolet UV domain, centered at approximately 380 nm and the other in the visible region in the range of 450–765 nm, and in the wide band gap of 3.37 eV with a high exciton binding energy of 60 meV, which allows excitonic transitions at room temperature [12]. Zinc oxide is considered very suitable for the production of a large variety of sensor and transducers since it is relatively bio-safe and a biocompatible material [13]. The specific optical and photochemical properties of ZnO are also used in a large variety of biomedical applications, such as antitumoral, antibacterial, antifungal, antioxidant, anti-inflammatory, and wound healing agents, including novel vectors in the fields of bio-imaging and drug delivery [14].

There are different approaches for the synthesis of the ZnO nanoparticles, which are mainly divided into three categories: the vapor, the solution, and the solid phase [15]. The type and the concentration of the defects generated in a ZnO material depend greatly on the preparation method and directly impact their optical, catalytic, and biological properties [7,16]. As a result, over the past several years, different synthetic methods for obtaining nanostructured ZnO have been proposed, such as (i) chemical vapor condensation, arc discharge, hydrogen plasma-metal reaction, and laser pyrolysis in the vapor phase; (ii) microemulsion, direct precipitation [15], hydrothermal synthesis, sol–gel processing [7,13,17], sonochemical [17], and microbial processes taking place in the liquid phase; and (iii) ball milling carried out in the solid phase. However, the classic methods to prepare ZnO nanomaterials involve the use toxic chemicals, intensive energy consumption, and, in the case of functional materials, time consuming multi step processes. Research in the last decades focused on the development of more sustainable methods for the synthesis and functionalization of nanomaterials, such as biosynthesis using natural products as reagents [18], reactions in supercritical fluids [19], and microwave or ultrasound-assisted synthesis [17,20].

Most of the green chemistry methods reported include ZnO synthesis using biological materials (e.g., plant extracts, vitamins, sugars, biodegradable polymers, and microorganisms) having significant reducing and stabilizing features [21]. In a typical biogenic route, the biological entity acts not only as a reducing agent (yielding NPs), but also as a capping agent, coating the NP surface, preventing their oxidation and degradation and enhancing their biocompatibility. In addition, depending on the nature of the biological entity used, an extra feature can be given to the NP’s surface, such as an improvement of its antimicrobial activity, compared to the NPs synthesized by conventional chemical or physical routes [22]. However, the reproducibility and control of the obtained nanopowders in those methods are difficult to achieve.

Recently, microwave-assisted synthesis has gained massive attraction owing to its advantages of simple equipment, low-cost, high safety factors, selectivity, fast heating speed, homogenous volumetric heating, high productivity, and low energy consumption compared to the other conventional heating methods [23,24]. The microwave–hydrothermal process enhances the crystallization kinetics because of the rapid and volumetric heating [25]. In addition, microwave heating processes are favorable in producing high-quality ZnO materials by minimizing contamination in grown ZnO crystals. In microwave processing, heat is generated internally within the materials, so an inverted temperature gradient occurs. The surfaces of materials are cooler than the interior and therefore the thermal losses are decreased [26]. After interacting with matter, microwaves can be reflected, passed or absorbed by the material. Polar molecules have molecular dipole moments that interact with the high frequency electromagnetic radiation. This interaction causes the molecules to vibrate and rotate which, in turn, causes the polar solution to heat up [27]. Hence, the enhancement in product formation and feasibility to tune morphology could make this synthesis process unique [28]. Microwave instruments have become more efficient and cheaper and are now increasingly being used as reactors for a wide variety of synthesis operations [29]. Previously, our group successfully employed this method to controllably grow flower-like ZnO nanostructures in the presence of *Saponaria officinalis* extracts [30].

Tremendous efforts have been made in recent years in the development of various organized micro or hierarchical ZnO with a variety of new morphologies. Quite a few interesting 3D structures of ZnO including flowers, prismatic tips, closed pine cones, bullets, nanobelts, nanobridges, nanonails, and nanoribbons [31] have been fabricated by hydrothermal, solvothermal, chemical vapor deposition, ionothermal, sol–gel, direct precipitation, microwave-assisted synthesis, etc. [10,32]. The growth of these differently shaped ZnO nanostructures largely depends on the relative stability of the growth and direction of the crystal faces [33]. A special category of ZnO NPs with very interesting properties is the one with flower-like morphology, which has applications in fields of textiles, sensors, cosmetics, storage, optical, and electrical devices [34].

Size, shape, and surface functionalization are key factors in the extent of the optical, electrical, and photocatalytic properties. Although the small size is preferable in most industrial applications because it leads to an increased surface area and surface reactivity, the high particle number-to-mass ratio becomes a disadvantage when considering aspects of toxicity and environmental impact. Although ZnO is considered to be non-toxic material and is approved for use in food packaging and functional textiles, recent research indicates increasing interest in the toxicity aspects of nanoparticulate ZnO. Cytotoxicity of ZnO NPs, as for other nanostructures with different composition, strongly depends on the size, shape, and surface coatings [35]. The metal and metal oxide NPs, such as Ag, Fe_3_O_4_, ZnO, CuO, and Ce_2_O with dimensions in the range 1–20 nm express in particular significant threats when interacting with the living organism due to specific accumulation in tissues, disturbing normal metabolism and function of the cells without signs of acute toxicity [36].

In most cases, functionalization with ZnO nanoparticles aims to obtain materials with antibacterial properties. In this sense, it is very important to carefully tune the size of the nanoparticles to ensure the highest possible antimicrobial efficiency but to keep the toxicity within low limits.

Thus, flower-like aggregates formed by small ZnO NPs have gained significant interest since those 3D nano/microstructures could combine the advantages of reduced sizes of component nanostructures with a lower toxic effect due to clustering.

Several types of syntheses have been proposed for obtaining flower-like NPs. The most commonly used are the hydrothermal route, controlled precipitation, spray pyrolysis, sol–gel method, chemical bath deposition, electro-deposition, and sonochemical methods. Biological methods (using microorganisms and plant materials) have gradually been established as simpler, more economical, and greener methods to synthesize flower-like ZnO nanoparticles, with the potential to replace the conventional methods. Moreover, the plant-assisted routes possess bioreducing and/or bio-template components such as reducing sugars, flavonoids, and proteins [37], which allow control of the particle’s size and morphologies. This should be very useful because the particle morphology can be tuned according to its final application. Adjusting the pH value, temperature, and the molar ratio of Zn^2+^ and capping agents has been found to have a significant influence on the morphology of ZnO nanostructures. Among them, the pH value is believed to be a key factor because it can greatly change the quantity of ZnO nuclei and growth units [38].

The impact of nanotechnology in the textile industry has made it possible to produce a new generation of textiles by innovative finishes of the fabric surface. The textile materials functionalized with nanostructures have proven to be useful for many applications, such as photocatalytic self-cleaning and purification, antimicrobial and ultraviolet (UV) light protection, flame retardancy, thermal insulation and moisture management, hydrophobicity, and electrical conductivity [39]. These “smart” textiles can be widely used for wound healing and medical applications in hospitals and other places where bacteria present a hazard. Recently, several nanomaterials, such as copper, gold, silver, aluminum, titanium dioxide, and zinc oxide (ZnO), have frequently been used in the textile industry as coating or embedding agents. However, the main problem that often accompanies the application of metal and metal oxide nanoparticles to textile materials via coating is agglomeration of the dispersed NPs. In order to control the deposition and dispersion of nanoparticles on the fabric surface, two methods are currently used for functionalization of the textiles. The first method is based on an ex situ procedure, where dispersion of nanoparticles is applied by dip coating, spraying, or immersion, followed by additional treatment with polymeric solutions as a stabilizing agent for finishing the coating on the textile [40]. The second method, known as in situ preparation, consists of binding the nanoparticles directly onto textile fabrics when the NPs are synthesized using the fabric as a template [41]. A facile variation of this in situ method, without stabilizing agents, uses the formation of a cavitation zone between two zinc electrodes in a high-power ultrasound reactor [42].

Further, diverse types of functional textiles, with antifouling photo-induced self-cleaning properties, have recently been developed, such as protein fibrous material (silk, fleece, feather, hair, etc.) and synthetic fibers (polyester, polyamide, activated carbon fiber, cellulosic fibers, cotton, linen, bamboo) etc. [43]. Although TiO_2_ is the most popular photocatalyst used in most multifunctional textile commercial products, ZnO is been also considered one of the most promising photocatalysts because of its outstanding properties, such as physical and chemical stability, low cost, and non-toxicity [43]. Due to its transparency for the visible wavelength range of sunlight, nanosized ZnO may be used as a blocker of harmful ultraviolet irradiation [44]. In addition, among various inorganic antimicrobial agents, zinc oxide (ZnO) has been verified to have good antibacterial activity against Gram-positive bacteria and Gram-negative bacteria and wound-healing properties [45]. It is well-known that ZnO NPs with a smaller size and larger concentration could achieve a better bactericidal response, and different methods have been developed to immobilize ZnO NPs onto fabrics.

ZnO NPs have other advantages. For example, compared to silver NPs, they have a lower cost and white appearance. Moreover, the antimicrobial activity of ZnO NPs is light-independent, in contrast to TiO_2_ NPs, where light irradiation is required for achieving good antibacterial activities [46].

The treatment of fabrics using ZnO NPs for antibacterial properties and improved UV radiation protection has been achieved by various methods including the “pad-dry-cure” method, radiation, homogeneous precipitation method, hydrothermal method, bio-approach, and layer-by-layer deposition method. In recent years, ultrasonic and microwave irradiation opened up new areas of the textile finishing process because it can dramatically reduce energy, chemicals, and the time involved in various operations [47].

The in situ deposition of ZnO nanoparticles on textile fibers may provide a simple, inexpensive, and green approach for scaled-up production of antibacterial fabrics [46]. This process involves in situ generation of ZnO and its subsequent deposition on fabrics in a one-step reaction [47]. This coating method is considered a “greener” approach because it does not involve supplementary hazardous materials in the deposition step. In addition, it was shown that even with a low coating concentration of ZnO in the composite (less than 1%), excellent antibacterial activity was achieved. The multiple parameters and different conditions involved during the synthesis process, for example, synthesis method, reagents used, pH of the medium, the temperature of the process, the processing time, concentrations of the precursors, reaction medium of the process, drying temperature, and time, influence the amount produced, structure and size of ZnO nanoparticles, as well as their performance and functional properties.

In the present work, a novel method to produce ZnO functionalizated textiles was investigated using microwave-assisted in situ synthesis with mild conditions and harmful reagents in order to decrease the effect on the fabric mechanical and optical properties. The possibility to obtain flower-like micro/nanostructures of ZnO strongly attached to the fabric was studied at relatively moderate temperatures and using a short period of microwave irradiation.

## 2. Materials and Methods

### 2.1. Materials

Zinc acetate dihydrate [Zn(CH_3_COO)_2_·2H_2_O] (powder, purity 98%), sodium hydroxide (NaOH) (pellets, purity ≥ 98%), arginine hydrochloride (C_6_H_14_N_4_O_2_·HCl) (powder, purity ≥ 98%), and ammonium hydroxide (NH_3_·H_2_O) (purity 99%) were all Aldrich reagents purchased from Sigma–Aldrich (Merck Group, Darmstadt, Germany) and used as received, without any additional purification. Ethanol 96% was purchased from ChimReactiv SRL (Bucharest, Romania). Bidistilled water was produced using a laboratory ultrapure water purification system (Milli-Q ^®^ Advantage A10, Merck Millipore, Germany).

### 2.2. Microwave-Assisted Synthesis of ZnO NPs and In Situ Deposition on Textile

Zinc oxide nanoparticles were fabricated using a method developed in our laboratory based on a combination of methods reported in the literature [23,25].

The synthesis was conducted via microwave heating from an ethanolic solution (water:ethanol = 1:1, *v*/*v*) of zinc acetate dihydrate [Zn(CH_3_COO)_2_·2H_2_O] and different co-reactants as reducing agents and pH regulators (ammonia solution, NaOH and Arginine). Concentrations of 2 mM and 20 mM of Zn(CH_3_COO)_2_·2H_2_O solution were used. In a typical synthesis, 10 mL of zinc precursor solution at desired concentration was stirred at room temperature until total dissolution. Then, the pH value of the solution was adjusted to 10–11 by adding NH_3_·H_2_O dropwise, under stirring, the solution first becoming opalescent, and then transparent when the pH value reached 11. A volume of 15 mL of mixture was subsequently transferred to a microwave reactor Monowave 200 (Anton Paar GmbH, Graz, Austria), into a microwave vial, rapidly heated at the target temperature (90 °C or 120 °C), and maintained at this temperature for a period of time under magnetic stirring. The heating step was performed with the “as fast as possible” option on the instrument setting. The magnetic stirring was ensured by the stirrer of the microwave reactor and was set at 1000 rpm during the reaction and cooling time. Similarly, samples using Arginine 20 mM solution, a mixture of Arginine/NaOH or Arginine/ammonia as reduction agents were prepared. After cooling, the obtained precipitates were separated by centrifugation and washed three times with an alcohol–water solution and then used without further thermal treatment.

For the in situ deposition of ZnO NPs on textiles, the synthesis was performed under the same conditions, with a (3 cm × 3 cm) fabric sample immersed inside the initial solution of the zinc precursor.

Commercially available cotton fabric was used as a model textile. The textile coupons were cleaned before experiments in a washing machine with 5% (*v*/*v*) solution of commercial detergent at 40 °C for 30 min followed by three rinsing cycles in distilled water, drying at ambient temperature, and then ironing.

### 2.3. Characterization

The as-prepared size and size distribution of ZnO NPs obtained in solution were determined using the dynamic light scattering (DLS) method, and the measurements were performed on a Zetasizer NanoZS instrument (Malvern Instruments Ltd., Malvern, UK). The measurements were carried out on pristine and diluted samples (the dilution was made with distilled water (1:10)), and samples were ultrasonicated for 5 min in an ultrasonic bath Branson 220 (Branson Ultrasonic Cleaner, Branson, Danbury, CT, USA).

UV-Vis spectra were recorded on a Jasco UV-Vis V630 spectrometer (Jasco, Easton, MD, USA) in the 200–900 nm range in a 1 cm quartz cell. Diffuse reflectance electronic spectra were recorded at room temperature with a Jasco UV-Vis-NIR V670 spectrometer (Jasco, Easton, MD, USA) in the 200–1500 nm range, with Spectralon as a reference standard. Fluorescence spectra of ZnO dispersions were recorded on a Jasco FP-6300 spectrofluorimeter (Jasco, Easton, MD, USA).

The morphology of ZnO nanopowders as a solid product or in situ deposited on textile was investigated through scanning electron microscopy (SEM). The images were obtained on an FEI Quanta 200 instrument (Eindhoven, The Netherlands) at 30 kV accelerating voltage, using a large field detector (LFD) and high vacuum (HV) working mode. The specimens for SEM investigations were prepared by casting a few drops from ZnO dispersions on aluminum stubs without further covering of the deposited samples. The ZnO coatings deposited on textile coupons were imaged using a fabric sample fixed on the microscope stub.

Powder X-ray diffractograms were recorded on a Shimadzu XRD-7000 diffractometer (Kyoto, Japan) using Cu Kα radiation (λ = 1.5418 Å, 40 kV, 40 mA) in the 10–90 degree 2θ range with a 2 degree/min scanning speed and a step of 0.2 degrees.

### 2.4. Photocatalytic Activity

The photocatalytic activity of synthesized ZnO NPs was evaluated by the degradation of Methylene blue (MB) at room temperature using solar light and low pressure mercury lamp as a radiation source. The optimum conditions for the experiment (initial concentration of dye, the loading of catalyst, time of stirring to achieve full adsorption on solid) were determined and reported in our previous study [30].

The initial solution containing the ZnO catalyst and MB (3mg/L) was stirred for 30 min in the dark to facilitate the adsorption of MB onto the semiconductor particle surface. Sample aliquots of 3 mL were extracted and monitored spectrophotometrically at λ = 616 and 664 nm in a quartz cell (1 cm). Concentrations of MB in solutions were determined using a calibration curve.

For the investigation of the photocatalytic ability of ZnO-functionalized textiles in the degradation of MB, 3 × 3 cm coupons of cotton were immersed in dye solution, and the degradation process was monitored as described above.

For the self-cleaning tests, coupons of untreated and ZnO modified cotton were stained using the same MB solution and left to dry for one day at room temperature in the dark in order to let the dye fully interact with the fabric. The surface of the stained textile samples was half covered with black paper and was then exposed to solar light (ambient environment outside of the university building, month of July) for 2 h, and the UV-Vis reflectance spectra on exposed and protected zones were recorded for each sample.

### 2.5. Antibacterial Activity

The antimicrobial assays were performed using standard microbial strains from the Microbial collection of the University of Bucharest, Faculty of Biology, Microbiology Department: *Staphylococcus aureus* ATCC 25923, *Escherichia coli* ATCC 25922, and *Candida albicans* ATCC 10231. For experimental assay, two successive passages on nutritious agar medium were performed followed by incubation for 24 h at 37 °C.

For the qualitative screening, an adapted spot diffusion method was followed (according with CLSI 2021 standard method). For the experiments, microbial suspensions of 1.5 × 10^8^ CFU/mL (0.5 McFarland density) were obtained from fresh cultures and were inoculated by spreading on the Petri dish agar medium. Subsequently, 2 cm^2^ sterile material specimens were disposed on the surface of the inoculated medium and incubated for 18 h at 37 °C. After 10 min, the plates were incubated for 16–18 h at room temperature. The positive results were read as the occurrence of an inhibition zone of microbial growth around the samples.

## 3. Results

### 3.1. Microwave-Assisted Synthesis of ZnO Nanoparticles

ZnO nanopowders were synthesized by using the microwave-assisted method described above. The sample coding and the reaction conditions are summarized in Table 1.

The zinc precursor concentration was chosen as 20 mM and 2 mM. For samples prepared in the presence of Arginine from solution Zn(Ac)_2_ with 2 mM concentration, a negligible amount of solid product was obtained; thus, no further investigations were performed on them.

The exposure to microwave irradiation was 5 and 15 min, and temperature was set as 90 and 120 °C according to our previous work [30].

Since the aim of the study is the in situ synthesis of ZnO NPs on the textiles, mild reaction conditions were targeted, from lower temperature to harmless reagents. The value of the pH was initially adjusted using ammonia solution and Arginine solution. As comparison, for the last samples (with Arginine), the influence of adding ammonia and NaOH as supplementary sources of hydroxyl ions was tested.

The ZnO nanopowders obtained at rather low temperature (room temperature up to 60 °C) required a long period of time for reaction development and led to the formation of solid products with low crystallinity, containing zinc compounds other than ZnO (such as Zn(OH)_2_ or zincite) [48]. Temperature exceeding 120 °C allowed the formation of ZnO nanopowders with high purity and crystallinity, but one can expect an increased impact on the fabric subjected to the in situ deposition. Thus, a temperature of 120 °C was selected as optimal for the in situ ZnO NP production on cotton samples.

Various reactants have been employed as basic media for ZnO precipitation, usually strong bases such as NaOH and KOH. In the present work a modified method with weak bases (ammonia solution and a model amino acid Arginine) was proposed in order to decrease the influence of the reagents on the textile sample.

In this case, the formation of ZnO was based on a hydrolysis reaction [49] according to the following equations:

Zn(CH_3_COO)_2_ → 2CH_3_COO^−^ + Zn^2+^ (1)

NH_3_ + H_2_O ↔ NH_4_^+^ + OH^−^ (2)

Zn^2+^ + 2OH^−^ → Zn(OH)_2_ (3)

Zn^2+^ + 4OH^−^ → Zn(OH)_4_^2−^ (4)

Zn^2+^ + 4NH_4_^+^ → [Zn(NH_3_)_4_]^2+^ (5)

(6) $$[\mathrm{Zn}({\mathrm{NH}}_{3}){}_{4}]{}^{2+}+2{\mathrm{OH}}^{–}\;\overset{\mathrm{microwave}}{\to }\;\mathrm{ZnO}+4{\mathrm{NH}}_{3}+H_{2}O$$

(7) $$\mathrm{Zn}{\left(\mathrm{OH}\right)}_{4}{}^{2–}\;\overset{\mathrm{microwave}}{\to }\;\mathrm{ZnO}+H_{2}O+2{\mathrm{OH}}^{–}$$

From the reaction between the zinc precursor and OH^−^ and NH4^+^ ions from ammonia, tetrahydroxozincate and tetraamine zincate were produced (Equations (4) and (5)). The dehydration of both complexes promoted nucleation and further transformation in ZnO nanoparticles.

The free amino group of the Arginine was responsible for the production of OH^−^ ions in aqueous solution, which further reacted with Zn^2+^ and promoted the formation of zinc hydroxides, as described above. The amino acid was supposed to act not only as a pH-adjusting agent and OH^−^ source but also as a directing agent due to the adsorption properties on the ZnO nuclei [50].

### 3.2. Characterization of ZnO NPs

The size and size distribution of the ZnO nanoparticles obtained under various conditions were determined in aqueous solution using the dynamic light scattering method (Table 2).

As expected, the concentration of reagents, temperature, and duration of microwave irradiation were factors that influenced the size and aggregation of ZnO nanopowders. In most cases, the ZnO nanoparticles exhibited a significant aggregation tendency, thus the DLS diagrams indicate a bimodal distribution of size, with large micronic aggregates present. When the samples were subjected to microwave irradiation for 15 min instead 5 min, (sample ZnO_Am4), two distinct population of particles could be observed in the samples. ZnO NPs with a smaller average size (143 nm for ammonia and 249 nm for Arginine/NaOH as pH modifying agents) were obtained, and in both cases large aggregates out of the range of the instrument were present, confirmed by the high value of the polydispersity index (PdI). The samples ZnO_Am4 and ZnO_Arg2 showed very high polydispersity (PdI = 1) and thus had a very broad distribution of particles, with large aggregates out of scale of the instrument, and the obtained DLS data are not reliable.

**Figure 1:**
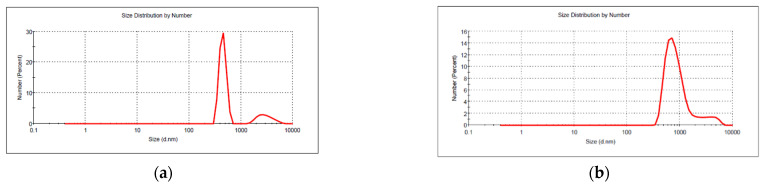
Size and size distribution of ZnO nanopowders obtained with ammonia (ZnO_Am2) (**a**) and Arginine/NaOH (ZnO_Arg1) (**b**), at 120 °C, 5 min microwave irradiation.

In Figure 1, representative DLS diagrams are shown for nanopowders prepared under optimized conditions (20 mM Zn^2+^ concentration, 5 min irradiation time, 120 °C temperature) in the presence of ammonia and Arginine/ammonia as pH-regulating agents.

The sample ZnO_Am2 (Figure 1a) prepared in the presence of ammonia showed a bimodal distribution, with the main population having an average size of 451 nm and a minor population of larger particles, with an average size of 3005 nm. The corresponding sample ZnO_Arg1 (Figure 1b) prepared under the same conditions but in the presence of Arginine/NaOH showed a similar bimodal distribution, with a population with an average size of 868 nm and the second one with an average size of 3989 nm. The higher value of the average size of nanoparticles obtained using NaOH as a pH-regulating agent is due to the kinetics of the processes in this case (i.e., slower nucleation rate compared to crystal growth, which is significantly faster [51]).

The sample ZnO_Arg3, where Arginine/ammonia mixture was used, had a monomodal distribution, with a single population with an average size of 2405 nm, without any additional signal indicating larger diffusional entities (Table 2). The lack of aggregates is due to the possible role played by the amino acid as a stabilizer.

**Figure 2:**
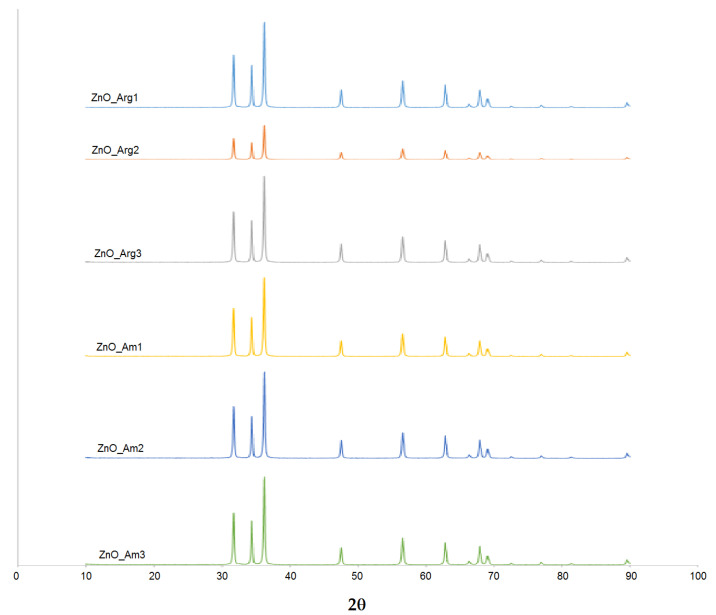
Representative XRD spectra of ZnO NPs prepared under various conditions. Details of encoding are summarized in Table 1.

The XRD patterns for all the samples (Figure 2) showed peaks at 31.74°, 34.38°, 36.22°, 47.54°, 56.58°, 62.82°, 67.96°, and 69.1°, which corresponded to the (100), (002), (101), (102), (110), (103), (112), and (201) crystal planes of the hexagonal phase of ZnO.

The diffraction peaks matched the wurtzite hexagonal phase pattern (JCPDS 36-1451). No additional peaks characteristic of impurities were present in spectra obtained for all samples, thus products with a high crystallinity degree and purity were obtained using the proposed microwave-assisted method. Furthermore, variation of the reaction conditions (time of irradiation, temperature) and reagents did not significantly affect the crystalline phase and purity of ZnO nanopowders. The size of the crystallites was estimated using Sherrer’s equation, with the most intense line at ~36° corresponding to the (101) plane. The size of crystallites was found to range from 35 to 42 nm, suggesting that the ZnO powder produced was polycrystalline.

**Figure 3:**
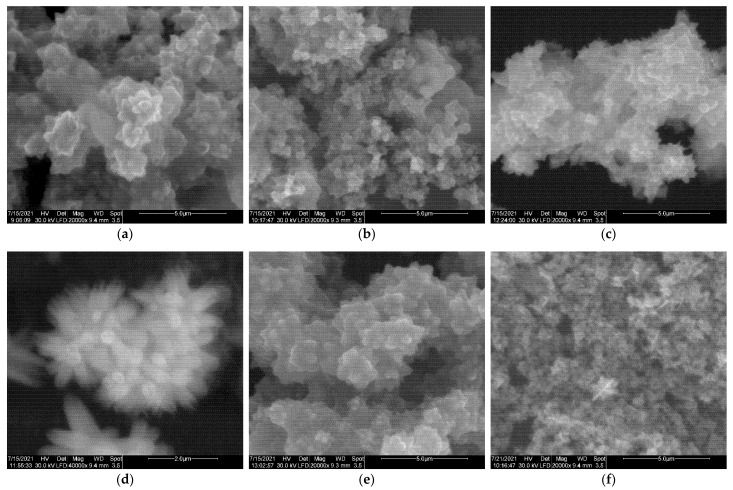

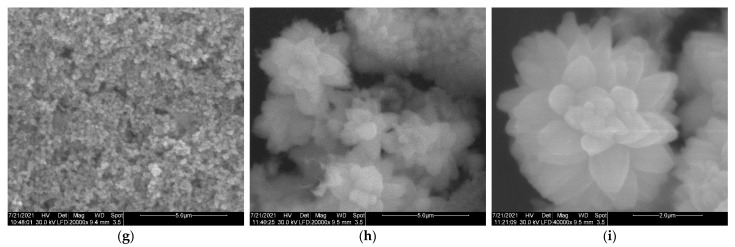
SEM images of ZnO nanoparticles obtained under various conditions (Table 1) in microwave-assisted synthesis. (**a**) ZnO_Am1; (**b**) ZnO_Am2; (**c**) ZnO_Am3; (**d**) ZnO_Am3 high magnification; (**e**) ZnO_Am4; (**f**) ZnO_Arg1; (**g**) ZnO_Arg2; (**h**) ZnO_Arg3; (**i**) ZnO_Arg3 high magnification.

Morphology of the obtained ZnO products was investigated using SEM (Figure 3). Figure 3a–e show images of flower-like microstructures obtained when ammonia was used as a pH-regulating agent. It can be observed that bundles resembling stars or a narcissus flower were formed in all samples of ZnO_Am. A single flower-like sample was composed of petals with a quasi triangular shape, with size in the range 300–800 nm, gathered in the center and forming 3D structures with a diameter ranging from hundreds of nm to 1 micron. For sample ZnO_Am3, prepared at a lower zinc precursor concentration, thus at a higher Zn^2+^/OH^−^ molar ratio, a different shape of petals was obtained (Figure 3d). Flower structures could be observed, but formed from hexagonal nanorods, with a diameter of a single rod of 200 nm and length of 500–800 nm. Unexpectedly, the sample ZnO_Arg2, prepared in the presence of Arginine (Figure 3g), exhibited a quasi-spherical shape of ZnO nanopowder, with a rather small diameter of particles, according to DLS results. The previously reported ZnO particles obtained in Arginine were found to be rod-like or dandelion flower-like, but the method used was solvothermal in an autoclave at high temperature (180 °C) and a much longer reaction time [52].

Figure 3f,h show the morphology of ZnO powders obtained in the presence of Arginine with the addition of NaOH and ammonia as supplementary OH^−^ sources. In the case of ammonia addition, flower structures such as narcissus-like previously described were obtained (Figure 3h and high resolution Figure 3i). In the samples prepared in the presence of Arginine/NaOH (Figure 3f), a mixture of spherical particles and flower-like aggregates formed from nanosheets with thicknesses of 40–90 nm could be observed.

ZnO nanomaterials exhibit remarkable optical properties, depending on their size, shape, and surface functionalization. ZnO shows strong absorption in the UV region, with a shoulder or maximum in the region of 350–400 nm due to the electron transition from the valence band to the conduction band. The presence of dopant impurities, various sizes of nanocrystallytes, and defects in the lattice produce shifts in the position of the specific absorption band of macrocrystalline ZnO, and a significant blue shift occurs when the size of nanoparticles decreases [53].

**Figure 4:**
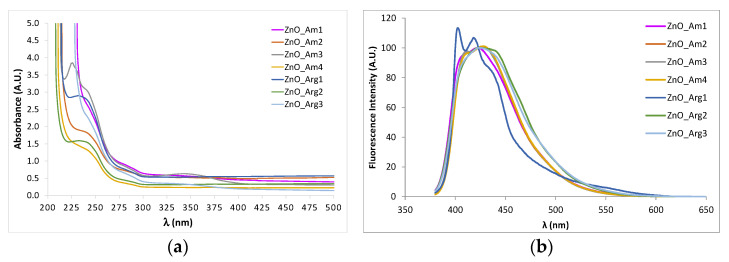
UV-Vis (**a**) and fluorescence (**b**) spectra of ZnO NPs prepared under various conditions using the microwave-assisted method.

In Figure 4, UV-Vis and fluorescence spectra of ZnO NPs are presented.

All samples prepared showed strong absorption in the wavelength region of 200–400 nm, which became negligible in the visible region. Specific peaks that can be assigned to the intrinsic band-gap absorption of ZnO in the region of 350 nm were present, with a different intensity, according to different sizes and morphologies. However, the shoulder in the absorption spectrum related to the transition to the exciton state did not show high energy since the size of the obtained ZnO particles was not very small.

Fluorescence emission of ZnO colloidal dispersions in aqueous media was measured in order to further investigate the optical properties (Figure 4b). Under excitation at 254 nm, a broad emission peak was observed for all samples with a maximum at 450 nm. For the emission in the green region exhibited by ZnO nanomaterials, several explanations were proposed, such as surface defects and various transitions between ionized oxygen vacancies and photoexcited holes or between electrons close to the conductive band and deeply trapped holes [54].

Another peak appeared in the blue-violet region, approximately at 380 nm in the spectra of sample ZnO_Arg1, prepared using both Arginine and NaOH as OH^−^ sources. This band of emission is due to exciton radiation in the near-field zone and corresponds to the interstitial defects of zinc.

### 3.3. Photocatalytic Properties of ZnO NPs

Photocatalytic properties of ZnO nanopowders were investigated for their ability to photodegradate methylene blue (MB) as a model organic dye under UV irradiation.

Pseudo-first-order constants, half-life times, and the photodegradation efficiency were calculated using the following formulas [30]:

ln(A_0_/A) = k_I_ t (8)

t_1/2_ = ln2/k_I_ (9)

% of degradation = [(A_0_ − A)/A_0_] × 100 (10)

where A_0_ is the absorbance of the solution at 664 nm before the irradiation, and A is the absorbance at a certain time of irradiation (t).

**Figure 5:**
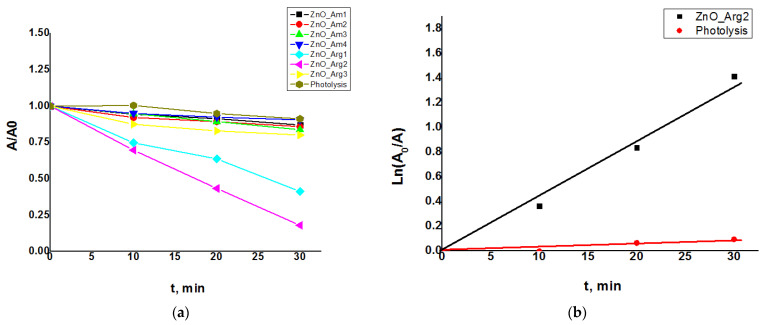
The discoloration curves of the MB with and without ZnO photocatalysts under UV irradiation. (**a**) Pseudo-first-order reaction kinetics in the absence of a catalyst and (**b**) in the presence of the ZnO_Arg2 sample.

In Figure 5, the results of the photodegradation of MB in the presence of ZnO as a catalyst are summarized for the samples prepared under various conditions.

The presence of ZnO nanopowders in the MB solution diminished the intensity of the absorption of the selected peak (664 nm) after the exposure to both UV and solar irradiation. The effect was significantly increased compared to photolysis in the absence of the ZnO nanomaterial. All the prepared samples exhibited enhanced photocatalytic activity with different intensities according to size and morphology. The best results were obtained for samples containing arginine ZnO_Arg1, ZnO_Arg2, and ZnO_Arg3, with a ratio A/A_0_ close to 0.18 for the sample ZnO_Arg2, which did not present flower-like 3D aggregates and had relatively reduced dimensions of particles. The samples prepared in ammonia (ZnO_Am1 ÷ ZnO_Am4) showed a similar discoloration ratio ranging from 0.8 to 0.9 due to their morphology of microstructures formed by clustering the ZnO nanoparticles.

Figure 5b compares the results obtained for pseudo-first-order constants in the absence of the ZnO photocatalyst and in its presence for the ZnO_Arg2 sample. The rate constant obtained in the presence of the catalyst ZnO_Arg2 (k = 0.0561 min^–1^ and t_1/2_ = 12 min) was significantly higher than that without the catalyst (k = 0.0024 min^–1^ and t_1/2_ = 288 min). The kinetics of the degradation process depend on both the shape and the size of the photocatalyst particles. For samples prepared with ammonia (ZnO_Am), a slower process of photodegradation of MB than for samples prepared in the presence of Arginine (ZnO_Arg) was observed, the half-life being in the case of the latter 10 times shorter.

In Table 3 are the kinetic parameters (pseudo-first-order constants and half-life times) for ZnO samples obtained with the microwave-assisted method.

Samples ZnO_Arg1, ZnO_Arg2, and ZnO_Arg3 showed a better catalytic activity and a short half-life (25 min, 12 min, and 59 min, respectively) for MB degradation compared with the other synthetized ZnOs. The pseudo-first-order rate constants decreased in the following order: ZnO_Arg2 > ZnO_Arg1 > ZnO_Arg3 > ZnO_Am2 > ZnO_Am3 > ZnO_Am1 > ZnO_Am4.

**Figure 6:**
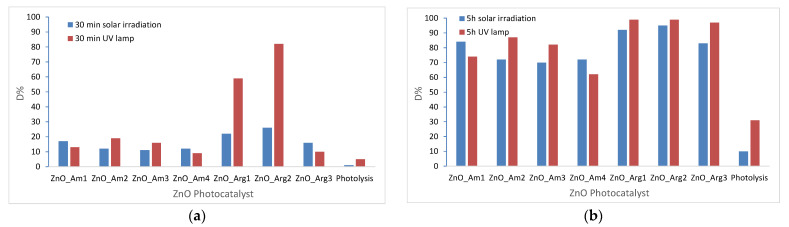
Degradation efficiencies of ZnO samples for MB photodegradation in the presence of artificial and solar light (**a**) after 30 min irradiation; (**b**) after 5 h irradiation.

For the same initial concentration of MB and catalyst, a study was performed in the presence of solar irradiation, and the results are presented in comparison with those artificially irradiated in Figure 6.

As can be observed in Figure 6a, by using artificial light, 82% of MB was degraded by ZnO_Arg2 and 59% by ZnO_Arg1, and by solar irradiation, 22% of MB was degraded by ZnO_Arg2 and 26% by ZnO_Arg1 after 30 min of irradiation. The other catalysts showed lower catalytic activity.

The longer exposure to the artificial and natural solar light produced for all samples a degradation of MB higher than 62% after 5 h, significantly higher than the effect of 10% reduction due to simple organic dye photolysis (Figure 6b). The samples prepared with ammonia (ZnO_Am) exposed to solar light exhibitd increasing photocatalytic efficiency in the following order: 70% ZnO_Am3, 72% both, ZnO_Am2 and ZnO_Am4, and 84% ZnO_Am1, according to their size and degree of aggregation in the flower-like structure as revealed by DLS and SEM data. Again, a higher percentage of MB degradation was obtained by using ZnO samples prepared with Arginine, up to 92 and 95% by solar irradiation and 99% by artificial irradiation for samples ZnO_Arg1 and ZnO_Arg2, respectively. Clearly, the degradation of MB under solar irradiation in the presence of a catalyst will need a longer exposure time in comparison to the irradiation with a low-pressure Hg lamp, but a prolonged irradiation time is not a problem, with the solar light being a free energy source and a non-pollutant alternative.

### 3.4. In Situ Synthesis and Characterization of ZnO NPs on Model Textiles

In order to obtain ZnO NP-based coating directly on the fabric, the previously described synthesis was performed under the same condition, with a coupon of the textile in the reaction media. The samples were similar to the ones in Table 1, with the T extension. The proposed method, using microwave irradiation, is supposed to be more efficient in terms of energy consumption and to promote stronger adhesion of the ZnO NPs to the fabric.

**Figure 7:**
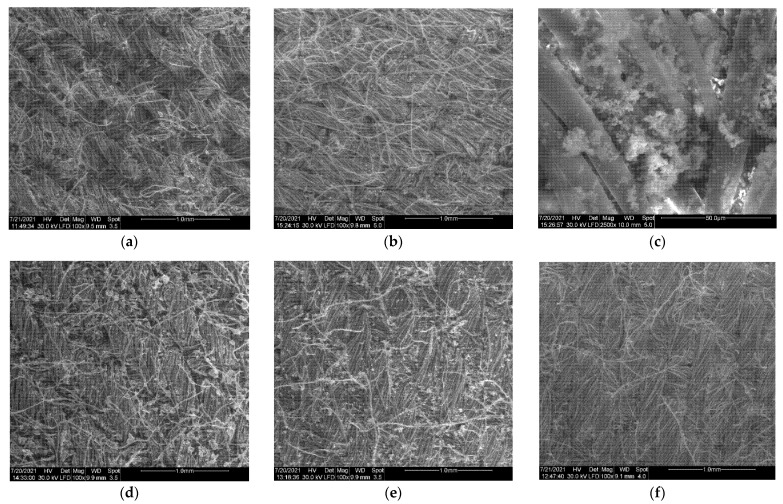

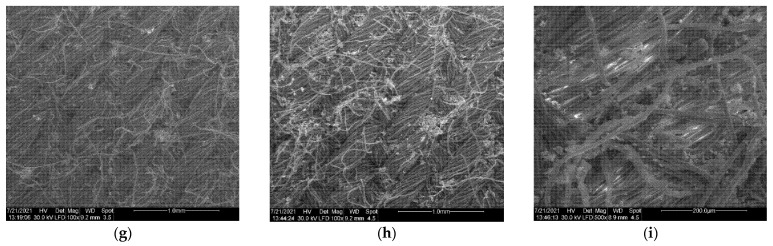
Surface morphology of cotton samples of coated with in situ synthesized ZnO NPs produced by the microwave-assisted method, under various conditions (*). (**a**) ZnO_Am1T; (**b**) ZnO_Am2T; (**c**) ZnO_Am2T high magnification; (**d**) ZnO_Am3T; (**e**) ZnO_Am4T; (**f**) ZnO_Arg1T; (**g**) ZnO_Arg2T; (**h**) ZnO_Arg3T; (**i**) ZnO_Arg3T high magnification. (*) Details on ZnO sample composition are summarized in Table 1.

The surface morphology of the cotton samples coated with in situ synthesized ZnO NPs was investigated by SEM, and representative images are presented in Figure 7.

The unmodified cotton coupon showed a smooth texture with visible fibrils. The growing of ZnO NPs on the fabric resulted in the presence of relative uniformly distributed particles on the whole textile surface, regardless of the condition of reaction. In general, a relatively dense and homogeneous coating of the ZnO particles can be realized by using the proposed method of in situ microwave-assisted synthesis.

The shapes of the ZnO nanopowders prepared in the presence of the textile were similar to those in the absence of cotton samples. For the ZnO_Am2T sample, prepared with 20 mM zinc acetate and ammonia, at optimized conditions, i.e., 5-min microwave irradiation and 120 °C, the flower-like 3D structures were present on the fabric, with the narcissus shape resembling the ones obtained in solution (Figure 7c).

In most cases, ZnO particles exhibited a tendency to aggregate on the surface of the textile material due to the limitation of the stirring possibilities in the microwave instrument used in the present experiment. In Figure 7b,c, for the sample ZnO_Am2T, large agglomerations of particles were detected, which is consistent with the result from DLS measurement that confirms the presence of aggregates in solution. Better results in terms of the distribution of nanoparticles attached to the fabric surface could be obtained using simultaneous microwave and ultrasonic treatment during the synthesis [20].

One could expect that the aggregation of the ZnO nanoparticles on the surfaces of the textile material will generate differences in the photocatalytic behavior of the ZnO nanopowders dispersed in solution and deposited onto the fabric.

**Figure 8:**
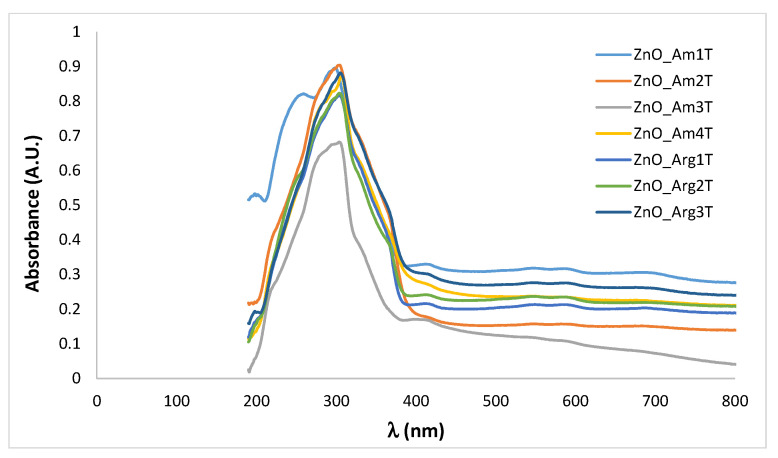
UV-Vis spectra of ZnO NPs prepared in situ on cotton under microwave irradiation under various conditions (*) on textile support. (*) Sample codification corresponding to the reaction conditions that are detailed in Table 1.

The optical properties of the cotton samples treated with ZnO nanopowders were assessed by UV-Vis spectroscopy in order to further evaluate the UV-protection efficiency of the functionalized fabrics. In Figure 8, UV-Vis reflectance spectra of the deposited ZnO nanomaterials on textile pieces during the in situ synthesis are presented.

For all cotton samples coated with ZnO NPs obtained under various microwave conditions, a strong absorption band, centered around 320 nm, is present, due to the strong UV absorbance of ZnO nanoparticles attached to the textile surface. Thus, the spectral results suggest that the successful coating of cotton samples could have high efficiency in absorbing ultraviolet light.

### 3.5. Photocatalytic, Self-Cleaning, and UV-Blocking Properties of ZnO-Functionalized Textiles

**Figure 9:**
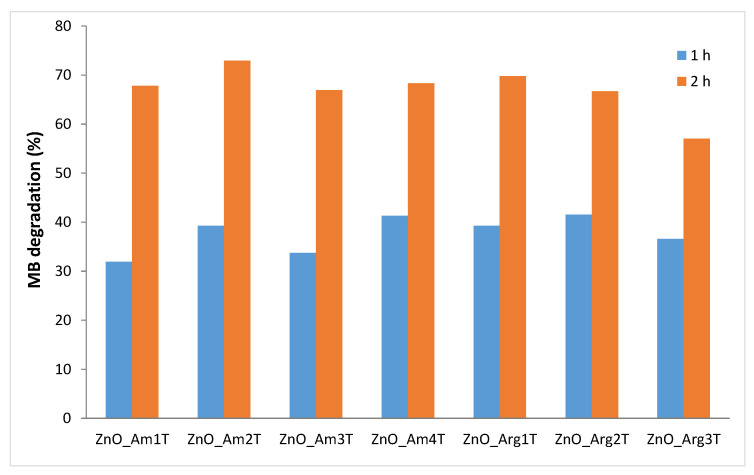
Efficiency of MB removal of ZnO-modified cotton samples. Reaction conditions are detailed in Table 1.

It is expected that the presence of the nanoparticulate ZnO coating formed during in situ synthesis on the cotton pieces resulted in the transfer of photocatalytic properties to the functionalized textiles. The ability of the treated cotton to act as a catalyst was monitored via the degradation of methylene blue (MB) in solution, exposed to natural light. The degradation of MB and thus removal from the solution, is considered from the decrease in the dye concentration assessed through the decrease in the specific absorbance at 665 nm. In Figure 9, the variation of MB removal with the ZnO coating obtained for fabrics under different conditions (microwave irradiation time, temperature, reagents) is presented.

After 1 h of exposure to natural sunlight, all modified textile samples exhibited a dye removal from 31.9% in the case of ZnO_Am1T up to 41.5% in ZnO_Arg2T. The experiments in the absence of the textile catalyst and in the presence of untreated sample, in the dark, under continuous stirring for 1 h were performed. The solar irradiation of the MB solution in the absence of modified cotton led to a dye removal of 19.8%, as expected for photolysis results for problematic organic dyes such as MB. A small amount of MB (12.7%) was removed from the solution kept in the dark, under stirring, due to the adsorption on the reference sample of untreated cotton. The extension of the irradiation time to 2 h led to an increase in the MB removal efficiency, with a maximum value of 72.9% shown by ZnO_Am1T, while the smallest value of 57% was recorded for the sample ZnO_Arg3T.

Some discrepancy with the photocatalytic properties for the bare ZnO NPs in MB solution was observed since in the case of the ZnO dispersions, the most efficient were samples prepared with Arginine (ZnO_Arg1 ÷ ZnO_Arg3), but deposited on textiles, high efficiency was shown by the ZnO_Am1 sample. The increase in the photocatalytic properties of sample ZnO_Am1 when the ZnO product was attached to the textile could be the result of clustering onto the surface of the fabric and the formation of large aggregates susceptible to easy detachment during the stirring procedure.

Considering the large amount of MB degraded in a relatively short period (2 h) of exposure to solar light observed in all cotton samples treated with ZnO NPs prepared in Arginine, the obtained functional textiles could be applied as reusable photocatalysts in the degradation of organic pollutants.

**Figure 10:**
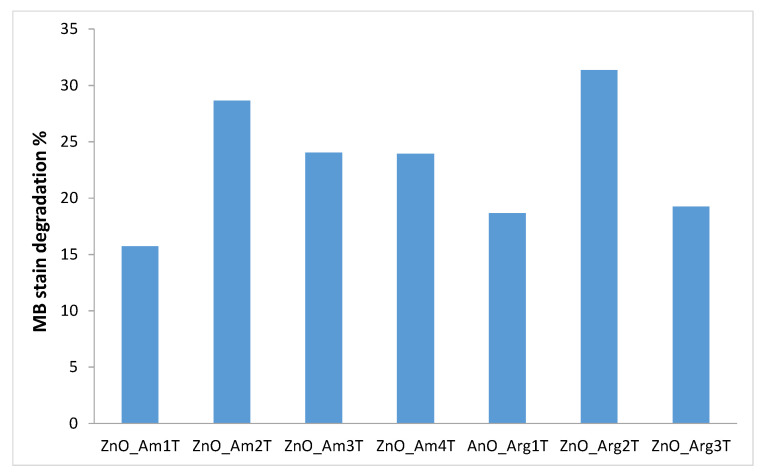
Discoloration of MB stains on the cotton samples functionalized with ZnO NPs obtained under various conditions.

The nanoparticulate ZnO-based coating deposited onto textiles could also confer self-cleaning properties. Figure 10 shows the visual aspect of textile pieces stained with MB as a model dye and the efficiency of discoloration on samples exposed to natural light.

The self-cleaning properties were expressed as a percentage of the absorbance of MB variation (changes in absorbance at the maximum absorption peak of 665 nm were assessed) on ZnO functionalized cotton when exposed to solar irradiation.

Cotton samples coated with ZnO_Arg2 and ZnO_Am2 exhibited destaining capacity up to 31% in terms of discoloration of MB adsorbed onto the textile after 2 h of exposure of solar light.

Degradation of MB resulted in lightening the color of the stain and formation of degradation products that are more likely to interact with detergents during washing procedures (see significant degree of stain cleaning on treated fabrics compared to reference unmodified cotton).

One of the most desired applications of ZnO-functionalized textiles is the fabrication of clothing with UV-shielding properties. In order to evaluate the UV-protective properties of the ZnO-treated cotton fabrics, UV absorption and transmission measurements were performed. As shown in the previous paragraph, the attachment of ZnO NPs on the textile by the proposed in situ method resulted in the decrease of UV radiation transmittance through treated fabrics for all types of obtained ZnO NPs.

The blocking percentages in the UV-A region (315–400 nm) and the UV-B region (280–315 nm) were computed according to the following equation [45]:

(11) $$\mathrm{UV}-A\;\mathrm{blocking}\;(\%)\;=\;\frac{\sum_{\lambda =315\mathrm{nm}}^{\lambda =400\mathrm{nm}}\;T(\lambda )\times \Delta \lambda }{\sum_{\lambda =315\mathrm{nm}}^{\lambda =400\mathrm{nm}}\;\Delta \lambda }\;100(\%)$$

where *T* is the transmittance at the selected wavelength λ.

For the UV-B blocking efficiency, a similar equation was used; the wavelength range to apply the computing was 200–315 nm. The results are summarized in Table 4 for the cotton samples coated with various ZnO NPs.

The commercial cotton used as a model fabric was modified with standard finishing reagents used in the textile industry; thus, the reference sample exhibited moderate UV-blocking properties. The transmittance was lower than that reported in the literature for pristine cotton. This difference may be due to the structure of the fabric and initial treatment of fabrics with additive chemicals.

The presence of ZnO NPs on the surface of cotton pieces decreased the transmittance in both UV-A and UV-B regions. For all ZnO NPs used in textile coatings, poor results were attained, with less than 70% blocking efficiency of UV-B radiation, while better results for blocking radiation in the UV-A region (83 to 95% blocking efficiency) were obtained. As expected from the photocatalytic measurements, the textile sample functionalized with ZnO_Am3, which is less efficient as a catalyst, also exhibited the lowest degree of UV-blocking capacity of 77%.

A very good efficiency in radiation shielding, both in the UV-A and UV-B region, was registered for the cotton modified with ZnO_Arg3, with 96% blocking efficiency in both cases. These results are consistent with the high photocatalytic activity proved by these sample both in dispersion and deposited on fabrics.

As reported in literature, ZnO nanoparticles show higher UV shielding ability compared to microparticles due to their high specific surface area and refractive index. Thus, the differences between the samples prepared under various conditions as described above could be explained in terms of shape and size variation due to the presence of different pH-regulating agents and variation of microwave reaction parameters. However, one must take into account in the design of the most suitable ZnO NPs for the functionalization of textile that the decrease of the nanoparticle size increases the optical and photochemical properties, but at the same time results in a significant increase in toxicity and a negative impact on the environment.

### 3.6. Antimicrobial Properties of ZnO Functionalized Textiles

The antimicrobial activity of textile samples was examined both through the qualitative and quantitative methods against *Staphylococcus aureus* ATCC 25923, *Escherichia coli* ATCC 25922, and *Candida albicans* ATCC 10231 microbial strains. The qualitative tests showed clear inhibition zones for samples ZnO_Am4T and ZnO_Arg3T against all tested strains and ZnO_Am2T, ZnO_Arg2T against *Escherichia coli* ATCC 25922 and *Staphylococcus aureus* ATCC 25923 only, while sample ZnO_Am1T showed a clear inhibition zone for the Gram positive *Staphylococcus aureus* ATCC 25923 strain only.

**Figure 11:**
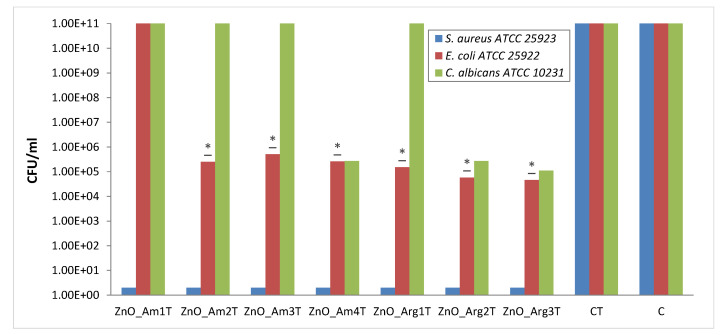
Graphical representation of the log10 values of colony forming units (CFU)/mL representing the viable cells of tested strains after the 18 h contact with tested samples; * 0.05 ≥ P(T ≤ t) > 0.001 significant evidence of inhibitory effect manifested by textile products on bacterial growth and multiplication. CT–control with untreated textile; C–control.

The qualitative results were confirmed by the quantitative test, the antibacterial assays suggesting that all textiles functionalized with ZnO NPs inhibited the growth of the three microorganisms, and the results depended on the tested strain and the size and morphology of the ZnO sample. All ZnO modified cotton samples, with the exception of ZnO_Am1, exhibited statistically significant antibacterial efficiency against the Gram negative bacterium *E. coli*, with a reduction of at least 4 logarithmic units in CFU/mL values compared to the growth controls (Figure 11). No influence on the growth of the eyeast strain *Candida albicans* ATCC 10231 was registered for textile samples decorated with ZnO_Am1 ÷ ZnO_Am3 and with ZnO_Arg1, while ZnO_Am4, ZnO_Arg2, and ZnO_Arg3 exhibited a good antimicrobial effect.

Exceptional antibacterial efficiency was obtained against Gram positive bacteria *S. aureus* when using all types of ZnO NPs, leading to the almost total inhibition of the microorganism growth.

To explain the antibacterial activity of ZnO nanopowders, several mechanisms have been proposed: (i) the release of Zn^2+^ ions, (ii) mechanical destruction of the cell membrane during the internalization of NPs, and (iii) ROS generation from the surface of the ZnO material [55].

Each mechanism was drastically influenced by the size, shape, and morphology of the ZnO particles. In particular, in the case of flower-like 3D aggregates, it is very difficult to compare the results reported in the literature for different morphologies, such as chrysanthemum-like, narcissus-like or rose-like ZnO structures. However, the results obtained in this study are consistent with other ZnO micrometric flower-like structures composed of “petals” with a size of 100–300 nm [56,57].

A further increase in antibacterial activity could be obtained through the addition of ultrasound treatment during the synthesis since it would significantly decrease the aggregation of particles [20].

The antimicrobial activity assessement proved that functional textiles prepared by in situ microwave-assisted synthesis showed efficiency in preventing microbial gowth of common strains and could be further improved as antibacterial fabrics.

## 4. Conclusions

A novel microwave-assisted synthesis of ZnO nanoparticles was developed for in situ deposition of particles on cotton fabrics. The single step procedure proved to be facile, with energy and time saving, and results in a relatively dense coating composed of ZnO flower-like 3D micro/nanostructures attached to the textile material. The SEM micrographs showed that in the presence of both ammonia and Arginine, as OH^−^ ion sources, flower-like aggregates of ZnO nanoparticles were obtained at a temperature of 120 °C after a very short exposure to microwave irradiation. The as-prepared ZnO nanoparticles exhibited significant photocatalytic properties in terms of model dye MB degradation in solution. The photocatalytic properties were transferred to the modified fabric when cotton substrates were decorated with ZnO during the in situ synthesis. The modified textile samples had good efficiency in MB degradation, varying from approximately 50% to more than 70% of MB removal after only 2 h of exposure to the solar light for all types of ZnO NPs. The best results were obtained for the series prepared with Arginine, with a maximum of 72.9% dye removal for the cotton with ZnO NPs with Arginine/NaOH. All textile samples functionalized with ZnO NPs prepared under various conditions showed self-cleaning properties and good UV protection efficiency.

The antibacterial activity was tested against common strains, *E. coli*, *S. aureus,* and *C. albicans*. In spite the relatively large size of the nanocrystallytes and 3D flower-like structures deposited on the textile, all ZnO functionalized samples exhibited an inhibitory effect on the tested strains. In particular, the most effective were the textiles decorated with ZnO nanoparticles prepared in Arginine, which exhibited a significant reduction of microbial growth in the case of *C. albicans*, with more resistant to the other samples.

The traditional procedure for fabric decoration with ZnO is usually a multistep method and requires rigorous conditions or sophisticated instrumentation. The synthesis proposed is simple and rapid, and further investigation to scale-up seems to be of great importance. The proposed eco-friendly method is a simple way to produce multifunctional textiles with photocatalytic and antibacterial properties as waste water purification support, self-cleaning, and UV-protecting materials.

## Figures and Tables

**Table 1:** ZnO NPs synthesized under various conditions.

Sample ID	Zn^2+^ Precursor Concentration (mM)	pH Modifier/ Stabilizer	Temperature (°C)	Time (min)
ZnO_Am1	20	NH_3_	90	5
ZnO_Am2	20	NH_3_	120	5
ZnO_Am3	2	NH_3_	120	5
ZnO_Am4	20	NH_3_	120	15
ZnO_Arg1	20	Arginine/NaOH	120	5
ZnO_Arg2	20	Arginine	120	5
ZnO_Arg3	20	Arginine/NH_3_	120	5

**Table 2:** Size and size distribution of ZnO NPs prepared under various conditions.

Sample ID	Size (nm)	PdI	Additional Peaks
ZnO_Am1	1781 ± 85.4	0.410	–
ZnO_Am2	451 ± 7.8	0.288	3049 ± 282.2
ZnO_Am3	1729 ± 34.1	0.226	–
ZnO_Arg1	868 ± 87.8	0.354	3989 ± 346.3
ZnO_Arg3	2405 ± 112.7	0.276	–

**Table 3:** Kinetic parameters for ZnO samples obtained under various conditions using the microwave-assisted method.

Sample ID	K_I_ (min^–1^)	t_1/2_ (min)
ZnO_Am1	0.0045	154
ZnO_Am2	0.00685	101
ZnO_Am3	0.00589	118
ZnO_Am4	0.00326	213
ZnO_Arg1	0.0281	25
ZnO_Arg2	0.0561	12
ZnO_Arg3	0.01176	59

**Table 4:** The UV blocking efficiency of ZnO-treated cotton.

Sample ID	UV-A Blocking (%)	UV-B Blocking (%)
ZnO_Am1T	83.63	64.76
ZnO_Am2T	86.30	64.49
ZnO_Am3T	77.32	52.77
ZnO_Am4T	84.70	69.54
ZnO_Arg1T	83.22	65.24
ZnO_Arg2T	83.65	65.52
ZnO_Arg3T	96.56	96.22
untreated cotton	48.85	34.83

## Data Availability

Not Applicable.
